# The problem of detrending when analysing potential indicators of disease elimination

**DOI:** 10.1016/j.jtbi.2019.04.011

**Published:** 2019-11-21

**Authors:** Adjani Gama Dessavre, Emma Southall, Michael J. Tildesley, Louise Dyson

**Affiliations:** a Mathematics Institute, University of Warwick, Coventry, UK; b School of Life Sciences, University of Warwick, Coventry, UK

## Abstract

As we strive towards the elimination of many burdensome diseases, the question of when intervention efforts may cease is increasingly important. It can be very difficult to know when prevalences are low enough that the disease will die out without further intervention, particularly for diseases that lack accurate tests. The consequences of stopping an intervention prematurely can put back elimination efforts by decades.

Critical slowing down theory predicts that as a dynamical system moves through a critical transition, deviations from the steady state return increasingly slowly. We study two potential indicators of disease elimination predicted by this theory, and investigate their response using a simple stochastic model. We compare our dynamical predictions to simulations of the fluctuation variance and coefficient of variation as the system moves through the transition to elimination. These comparisons demonstrate that the primary challenge facing the analysis of early warning signs in timeseries data is that of accurately ‘detrending’ the signal, in order to preserve the statistical properties of the fluctuations. We show here that detrending using the mean of even just four realisations of the process can give a significant improvement when compared to using a moving window average.

Taking this idea further, we consider a ‘metapopulation’ model of an endemic disease, in which infection spreads in various separated areas with some movement between the subpopulations. We successfully predict the behaviour of both variance and the coefficient of variation in a metapopulation by using information from the other subpopulations to detrend the system.

## Highlights

• We derive indicators of disease eradication so that control efforts may cease.
• Detrending is necessary to analyse single timeseries data and is difficult to achieve.
• Detrending using the mean of even a few simulations of the same process works well.
• Metapopulation models suggest a promising solution to the problem of detrending.

## 1 Introduction

The battle against infectious diseases includes some notable success stories, including the global eradication of smallpox (Fenner, 1982) and wild poliovirus type 2 (Adams and Salisbury, 2015), while the global mortality rate for malaria fell by 60% between 2000 and 2015 (The World Health Organisation, 2015). The 2012 London Declaration on neglected tropical diseases (NTDs) built on these successes by establishing goals for elimination and eradication of 10 NTDs by 2020. The intention of this declaration is to achieve elimination through various active interventions, from vector control to mass drug administration. The interventions aim to reduce the prevalence of the disease to such low levels that it is no longer sustainable, and dies out. However, each elimination program shares one fundamental challenge: how do we know when we can relax control? In this paper, we propose the use of statistical indicators derived using the theory of critical slowing down to assess how close the system is to eradication.

The theory of critical slowing down observes that in a dynamical system close to a critical transition, the rate of recovery from perturbations reduces. This is because as a steady state changes stability the real part of the dominant eigenvalue must pass through zero. Since real world systems are subject to noise, this phenomenon can be detected indirectly from an increasing “memory” in stochastic fluctuations, resulting in changes in statistical indicators (or early warning signs, EWS) such as variance and autocorrelation (Scheffer et al., 2009).

Indicators of critical transitions have been investigated in many areas, including the collapse of ecological systems (Carpenter, Cole, Pace, Batt, Brock, Cline, Coloso, Hodgson, Kitchell, Seekell, Smith, Weidel, 2011, Chen, Sanchez, Dai, Gore, 2014, Dakos, Bascompte, 2014, Drake, Griffen, 2010), the prediction of epidemic outbreaks (O’Regan and Drake, 2013) and changes in cellular populations (Dai, Korolev, Gore, 2015, Dai, Vorselen, Korolev, Gore, 2012). While these indicators have successfully predicted transitions in data (Dai, Korolev, Gore, 2015, Dakos, Scheffer, van Nes, Brovkin, Petoukhov, Held, 2008) and model simulations (Dakos, Carpenter, Brock, Ellison, Guttal, Ives, Kéfi, Livina, Seekell, van Nes, Scheffer, 2012, Held, Kleinen, 2004), potential indicators typically perform well for some models and poorly for others (Scheffer et al., 2012). A previous study deriving indicators of disease emergence and elimination was undertaken by O’Regan and Drake (2013), followed by the same authors investigating indicators of elimination in a vector-borne model (O’Regan et al., 2015). These two studies represent some of the first attempts to analytically derive the behaviour of potential indicators of critical transitions, rather than simply simulating a model (or looking at data) and observing the results.

Following these initial analytical papers, O’Regan and Burton investigated how the type of stochasticity included influences the behaviour of potential indicators of critical transitions (O’Regan and Burton, 2018). The authors showed that for their models the variance around the deterministic endemic steady state increases when additive stochasticity is included, but may decrease when including intrinsic noise. This indicates that the dominant noise type may affect the qualitative behaviour of indicators of critical transitions. In contrast, Sharma et al. (2016) found that the variance predicted well with both additive and multiplicative noise (and indeed with correlated noise), while autocorrelation predicted less well for multiplicative noise, noting that the autocorrelation, which would be predicted to rise, may instead be seen to decrease due to measurement noise.

Further work has been undertaken on disease emergence, addressing the difficulty of detrending seasonal infections (Miller et al., 2017) and comparing predictions with “imperfect” aggregated synthetic data, to investigate how EWS might behave with aggregated case reports at a weekly, bi-weekly and four-weekly rate (Brett et al., 2018). Seasonal forcing was found to decrease the predictive power of wavelet reddening, while the mean, variance, autocovariance and wavelet filtering did not significantly reduce in predictive power (Miller et al., 2017). In addition, removing the periodic trend was not found to improve prediction uniformly among statistics, and the authors conclude that seasonal detrending is often disadvantageous when using early warning signals. Reassuringly, Brett et al. (2018) found that most indicators can still predict disease emergence, even when the data are aggregated and subject to reporting errors, provided that the reporting error is not highly overdispersed. These results are mostly in accordance with (Frossard et al., 2015) who previously concluded that standard deviation and autocorrelation were robust to data aggregation, although skewness and kurtosis were found to perform poorly.

A number of experimental studies for ecological systems have shown that the changing characteristics in the spatial structure of the ecosystem can provide additional information when anticipating the approach to a critical transition (Dai, Korolev, Gore, 2013, Dakos, Kéfi, Rietkerk, van Nes, Scheffer, 2011, Dakos, van Nes, Donangelo, Fort, Scheffer, 2010, Guttal, Jayaprakash, 2009, Kéfi, Rietkerk, Alados, Pueyo, Papanastasis, ElAich, De Ruiter, 2007, van Belzen, van de Koppel, Kirwan, van der Wal, Herman, Dakos, Kéfi, Scheffer, Guntenspergen, Bouma, 2017). Following these studies (Kéfi et al., 2014) developed a statistical toolbox for implementing indicators of EWS, taking into account spatial heterogeneity. This toolbox provides a method pathway to determine the appropriate spatial indicator for a given dataset. Recent theoretical exploration by Chen et al. (2019) presents a novel spatial indicator that does not depend on the spatial mean and so bypasses the difficulty of detrending data. They suggest monitoring the dominant eigenvalue of the covariance matrix which was observed for ecological systems to rapidly increase on the approach to a transition and has the additional benefit that it could be used to identify spatial regions which are most vulnerable to a critical transition. This would be particularly informative for implementing control policies which target the highest risk areas.

The main aim of the work presented here is to derive indicators of elimination that may be of practical use in determining when an intervention may cease. This work highlights the importance and difficulty when calculating indicators for a single time-series of infectious case data, rather than over multiple realisations. There are two potential problems: since the theory considers fluctuations away from the mean, it is usually necessary to attempt to remove the mean to obtain the fluctuations. This is known as ‘detrending’ the data, and can be difficult to achieve in a single time-series. In addition, for a single time-series we cannot calculate ‘true’ statistics for the system but must instead use a moving window to approximate these. We show that detrending can give results that differ from those predicted, while the use of a moving window does not seem to present a problem. However, detrending using the mean of even a small number of simulations gives much improved results, suggesting a use for metapopulation models, where different geographical areas can be modelled as distinct subpopulations. We present a method for detrending such spatially collected data in order to calculate statistical indicators for a single outbreak using a metapopulation framework and analytically derive the covariance matrix for the spatial model to verify this. We show that these analytical predictions are similar to those found for a single simulation of the system, and can be used to determine whether a system is approaching extinction or not.

We note that not all diseases approaching elimination will necessarily go through a critical transition of the kind assumed here. Systems going through purely noise-induced transitions may present different indicators, and a rapid and nonlinear approach towards the transition may hinder the detection of a gradual trend (Boettiger and Hastings, 2012).

## 2 Analysis of a single population model

To investigate how EWS may be found in models of disease eradication, we begin by considering a very simple model: the classical SIS (Susceptible-Infected-Susceptible) model (Anderson and May, 1991). We study the slowly forced version of the model proposed by (O’Regan and Drake, 2013) where the transmission rate $\beta \left(t\right)=\left(1-pt\right)\beta_{0}$ is gradually reducing through time, until the disease is unsustainable. We consider the model in a stochastic formulation, with transition probabilities given in Table 1. This model is not intended here to represent any specific disease, but instead to allow us to develop our techniques on an analytically tractable system.

**Table 1:** Transition probabilities for the SIS model.

Event	Change in state	Transition rates
Infection		
Into *I*	I−1→I	$\frac{\beta (t)N-(I-1)(I-1)}{N}$
Out of *I*	I→I+1	$\frac{\beta (t)(N-I)I}{N}$
Removal		
Into *I*	I+1→I	γ(I+1)
Out of *I*	I→I−1	*γI*

We may analyse this model using the following master equation for *P*(*I, t*) (see (Gardiner, 2015), for example), where the probability of having *I* infectives at time *t* is given by

(1) $$\begin{array}{ccc} \frac{dP(I,t)}{dt} & = & -\left(\frac{\beta (t)(N-I)I}{N}P\left(I,t\right)-\gamma IP\left(I,t\right)\right)\\ & + & \frac{\beta (t)N-(I-1)(I-1)}{N}P\left(I-1,t\right)\;+\;\gamma \left(I+1\right)P\left(I+1,t\right). \end{array}$$

For this model the basic reproductive ratio at time *t* is $R_{0}=\beta \left(t\right)/\gamma$ (Anderson and May, 1991). For *R*_0_ > 1 the equivalent deterministic model admits a stable endemic equilibrium at $I/N=1-1/R_{0}$ while for *R*_0_ < 1 the infection dies out. As time increases, *β*(*t*) reduces in our model until *R*_0_ < 1 and the disease is unsustainable and dies out.

### 2.1 Analysis of fluctuations

Since the theory of critical slowing down relates to the fluctuations of the model about a steady state, we will use the linear noise approximation (Van Kampen, 1992), which assumes that

(2) $$\frac{I}{N}=\phi \left(t\right)+\frac{1}{\sqrt{N}}\zeta .$$

This results in a deterministic equation describing the mean system behaviour

(3) $$\frac{d\phi }{dt}=\beta \left(t\right)\phi \left(t\right)\left(1-\phi \left(t\right)\right)-\gamma \phi \left(t\right),$$

along with a linear Fokker–Planck equation (see supplementary information Section 1 for derivation), describing normally distributed fluctuations around this mean:

(4) $$\begin{array}{ccc} \frac{\partial \Pi }{\partial t} & = & -\frac{\partial }{\partial \zeta }[\left(\beta \left(t\right)\left(1-2\phi \left(t\right)\right)-\gamma \right)\zeta \Pi \left(\zeta ,t\right)]\\ & & +\;\frac{1}{2}\frac{\partial^{2}}{\partial \zeta^{2}}[\left(\beta \left(t\right)\phi \left(t\right)\left(1-\phi \left(t\right)\right)+\gamma \phi \left(t\right)\right)\Pi \left(\zeta ,t\right)]. \end{array}$$

There is little work in the literature undertaking a mathematical analysis of a particular model with the aim of predicting how indicators behave as a threshold is reached. Research that does make these predictions usually makes a steady state assumption at this point, allowing the straightforward calculation of potential indicators such as variance, lag-1 correlation and the coefficient of variation (O’Regan, Drake, 2013, O’Regan, Lillie, Drake, 2015, Brett, Drake, Rohani, 2017), and often plots these predictions against simulations taken at steady state. However this masks an important difficulty in the analysis of transition indicators, namely that systems undergoing transition are clearly not at steady state.

From Eq. (4) we may derive the variance of *ζ*(*t*), which satisfies

(5) $$\frac{\partial {\langle \zeta^{2}\rangle }_{t}}{\partial t}=2\left(\beta -\gamma -2\beta \phi \right){\langle \zeta^{2}\rangle }_{t}+\beta \left(1-\phi \right)\phi +\gamma \phi .$$

Using the linear noise approximation, it follows that the variance *V*(*t*) of the fluctuations around the mean system behaviour is $V\left(t\right)=\frac{1}{N}{\langle \zeta^{2}\rangle }_{t}$ and, at steady state, we obtain

$$V=\frac{1}{N}\frac{\gamma }{\bar{\beta }}=\frac{1}{N}\frac{1}{R_{0}},$$

where $\bar{\beta }$ is the value of *β* being used. However, we may also calculate from Eqs. (3) and (5), the time-varying solutions *ϕ*(*t*) and *V*(*t*). Similarly we may derive the coefficient of variation as $CV=\sqrt{V}/\phi$ as either a time-varying or steady state solution.

We compare numerical solutions of the variance and coefficient of variation using Eqs. (3) and (5) to simulated statistics (using the Gillespie algorithm (Gillespie, 1977)) of the underlying model (Fig. 2). In each figure we simulate three different stochastic systems. The first we denote Ext (extinct): the SIS system described by the transitions in Table 1, with $\beta \left(t\right)=\beta_{0}\left(1-pt\right)$ decreasing over time to zero at the end of the simulation. The second simulation is NExt (not extinct): as in Ext, but *β*(*t*) stops decreasing when it reaches 1.3*γ* so that $R_{0}=1.3$ for the rest of the simulation. Thirdly, we simulate FBeta (fixed beta): where $\beta \left(t\right)=\beta_{0}$ for all *t*. Parameter values used for the simulations are given in Table 2, and simulated data were given at timepoints 0.1 years apart.

**Table 2:** Simulation parameter values.

Parameter		Value
Initial transmission rate	*β*_0_	1 year${}^{-1}$
Recovery rate	*γ*	0.2 year${}^{-1}$
Change in transmission	*p*	1/500 year${}^{-1}$
Population size	*N*	20,000
Initial number of infections	*I*_0_	0.8*N*

### 2.2 Detrending simulations

**Fig. 1:**
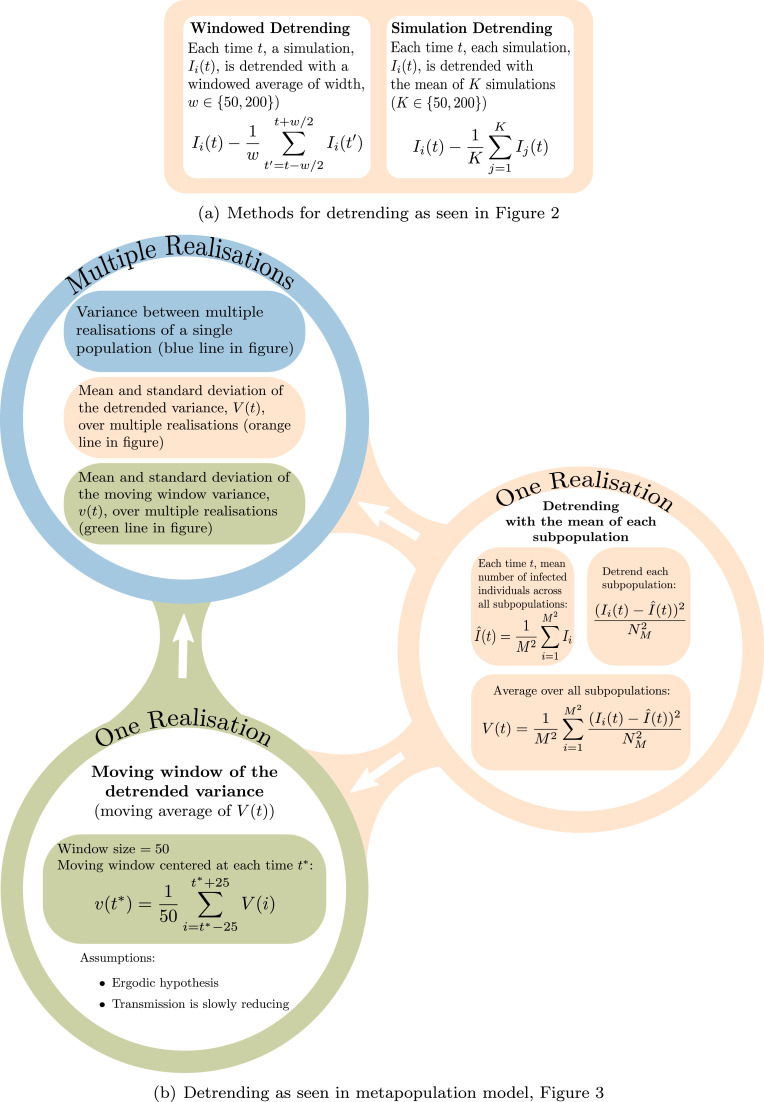
(a) Methods for detrending implemented for the SIS model. Left: detrending by calculating the mean over a window for one realisation (Windowed Detrending, as seen in Fig. 2 (b)). Right: detrending calculated over a subset of multiple realisations (simulation detrending, as seen in Fig. 2 (c) and (d)). (b) Methods for detrending and calculating the variance over *M*^2^ subpopulations, each with population size of $N_{M}=N/M^{2}$. This was implemented in Fig. 3. Each method was calculated over multiple realisations to assess the mean behaviour.

In order to interpret any potential indicators of elimination, simulations need to be carefully detrended. Detrending is required to remove long-term trends in the data in order to observe critical slowing down in the fluctuations. Typically, simulations are detrended over multiple realisations of the same process (simulation detrending, see Fig. 1(a)), however this is not applicable with real-world data. To calculate statistics from real data, in the EWS literature windowed detrending is usually used, where the moving window average is removed from the timeseries (windowed detrending, see Fig. 1(a)).

**Fig. 2:**
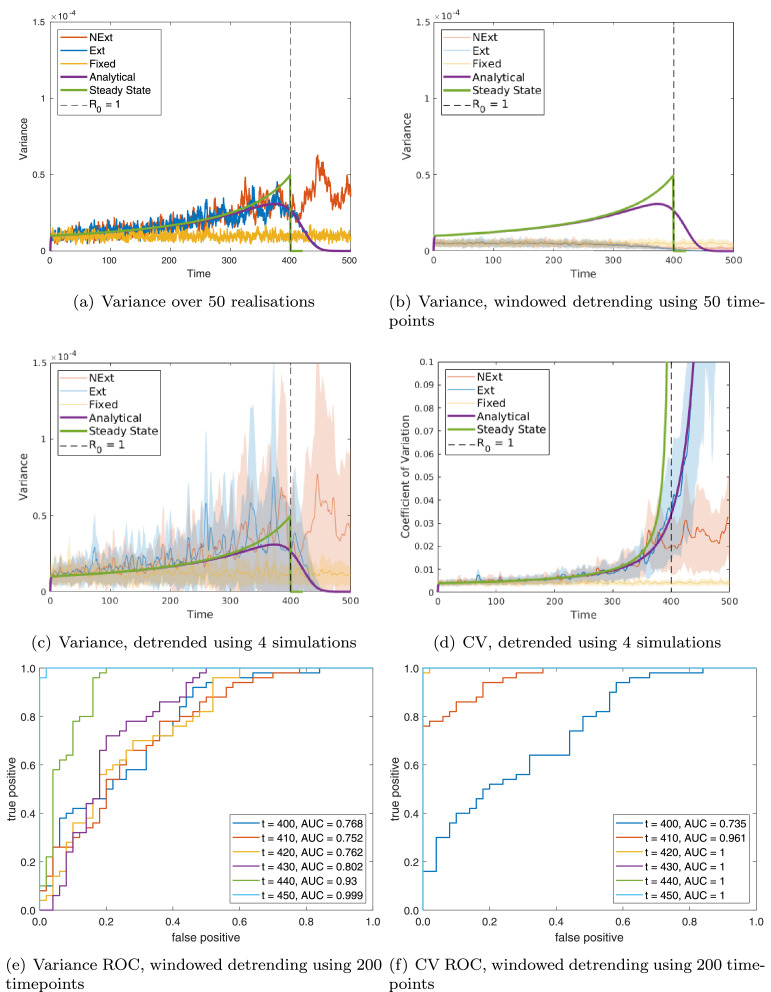
**Single population**: comparing predictions to simulations for variance: (a) over 50 realisations; (b) over a moving window of size 50 timepoints; and (c) over a moving window of size 50 timepoints, first detrending using the mean of 4 simulations and (d) of the CV calculated over a moving window of size 50 timepoints, first detrending using the mean of 4 simulations. ROC curves are calculated over 50 realisations at various timepoints by thresholding in (e) variance; and (f) CV using windowed detrending. For each ROC curve the legend gives the area under the curve (AUC), suggesting how predictive that indicator is (AUC closer to 1 are more predictive). Each figure shows: steady state predictions (green line); dynamic predictions (purple line); simulations of the model going extinct (Ext, blue line); simulations of the model not going extinct (NExt, red line); and simulations of the model with fixed *β* (FBeta, yellow line). For repeated simulations each line is the mean value obtained over 50 simulations and the shaded area represents one standard deviation about the mean. (For interpretation of the references to colour in this figure legend, the reader is referred to the web version of this article.)

**Fig. 3:**
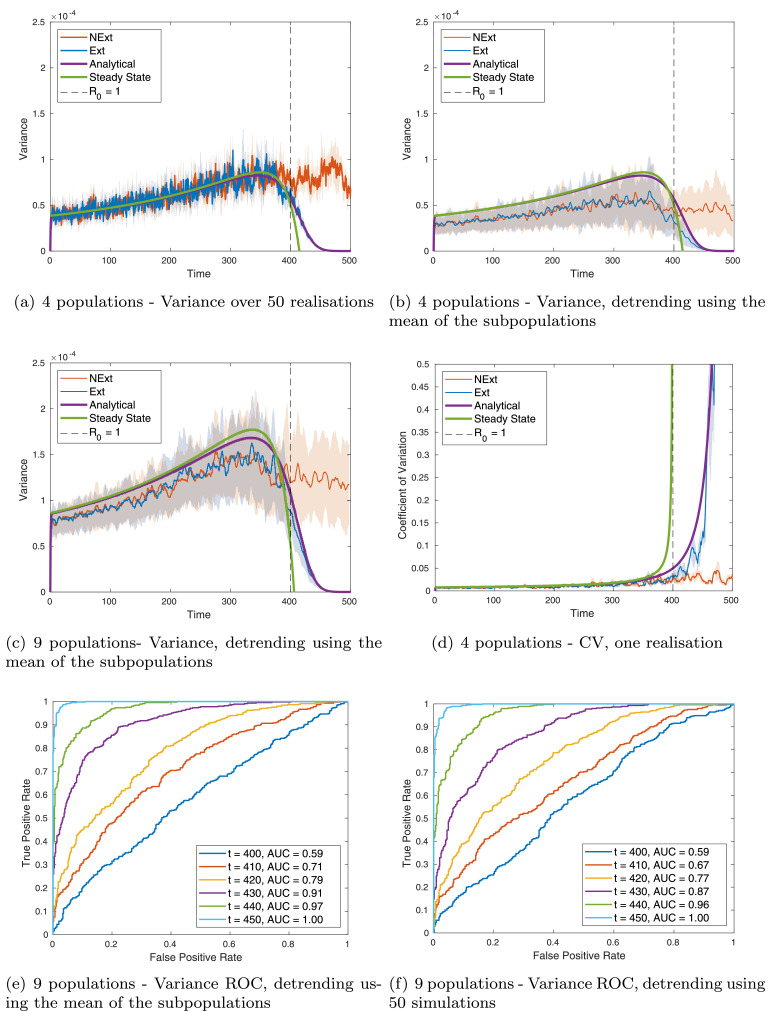
**Metapopulation:** comparing predictions to simulations for variance: (a) for M=2, over 50 realisations; (b) for M=2, over a moving window after detrending using the mean of the subpopulations; (c) for M=3, over a moving window after detrending using the mean of the subpopulations, (d) for CV, M=2, over a moving window after detrending using the mean of the subpopulations; (e) ROC curve calculated over 50 realisations at various timepoints by thresholding in variance, detrending using the mean the subpopulations; and (f) ROC curve calculated over 50 realisations at various timepoints by thresholding in variance, detrending using the mean of 50 realisations. Timeseries figures show: simulation of NExt (red line); simulation of Ext (blue line); dynamic predictions (purple line); steady state predictions (green line). For repeated simulations each line is the mean value obtained over the simulations and the shaded area represents one standard deviation about the mean. Note that for ROC curves no simulation had reached eradication at t=450, although all Ext simulations had achieved eradication by the end of the simulation. (For interpretation of the references to colour in this figure legend, the reader is referred to the web version of this article.)

Having detrended the data we may calculate each statistic in two ways: either using multiple simulations and finding the variance, say, between the realisations; or taking moving windowed statistics, assuming that the system is changing slowly enough to be approximately ergodic, so that the time-averaged statistic approximates the desired value. When considering real data we would take a moving windowed approach for both detrending and calculating the statistic. Thus there are essentially three approximations that are being made to the system: the linear noise approximation assumes gaussian noise and approximates integer numbers of infections by a continuous variable; the detrending of the signal; and the calculation of the moving window statistics.

### 2.3 Results for the single population model

#### 2.3.1 Variance

Our prediction for the variance (from Eq. (5)) is similar to the steady state values at early times, but increasingly diverges as we approach the transition (Fig. 2(a)). This is to be expected, as the theory of critical slowing down predicts that deviations from the steady state values return increasingly slowly as we approach the transition. Thus closer to the transition, the system does not react quickly enough to the changing parameter value to be able to reach the steady state. We can see from this that making predictions from the steady state theory is inherently problematic in a system that is undergoing even gradual parameter changes.

When we simulate the system multiple times and plot the variance between these simulations at each timepoint, we find that our predicted variance compares well with the Ext simulations (Fig. 2(a)). In comparison the variance of FBeta remains constant over time at the initial value, while NExt rises similarly to Ext to start with, before remaining at the higher level after *β* is held constant. This demonstrates that our analysis has successfully predicted the time-varying variance for this system.

In Fig. 2(b) we implement the windowed detrending for each realisation of our simulations and plot the resulting mean and confidence intervals. It is clear from this that the windowed approach can significantly change even the qualitative form of the obtained curves. In particular we observe no increase in variance in Ext or NExt, and instead see a gradual decline, levelling off for NExt, and continuing to zero in Ext. Unfortunately, this approach requires a judgement as to the appropriate size of the window: too big, and the ergodic assumption will break down; too small, and there simply isn’t enough data in the window to give a reliable estimate of the statistic. This can be clearly seen in the FBeta curve in Fig. 2(b), which does satisfy the ergodicity condition, but is significantly lower than predicted. Taking a larger window rectifies this problem (see supplementary figure S1(a)), but increasing the window can lead to spurious responses in Ext and NExt (result not shown).

In fact these undesirable results come about because of the detrending required prior to determining the windowed variance. If we detrend using the mean of many simulations, (or using predicted mean derived in Eq. (3)) then we obtain very similar results for a wide variety of window sizes, and these are very close to the predicted variance (see supplementary figure S1(b)). This shows that of the three previously mentioned approximations, it is the detrending that is causing problems and not the linear noise approximation or the windowed calculation of the statistics. The method of detrending is thus very important and, unfortunately, it is also very difficult to do well. The difficulty lies in assessing the extent of smoothing that is required. If the data is ‘overfitted’ then the detrended variance is significantly reduced, but if the trend is too smooth then this generates local spikes in the statistics that are not in response to thresholds. If we detrend using the mean of even a small number of repeated simulations, this can work very well (Fig. 2(c)), giving hope for the use of data from multiple similar areas, either for use in detrending, or for use in spatial statistics (Kéfi, Guttal, Brock, Carpenter, Ellison, Livina, Seekell, Scheffer, Van Nes, Dakos, 2014, Dai, Korolev, Gore, 2013).

#### 2.3.2 Coefficient of variation

It has been noted before that the coefficient of variation (CV, the standard deviation divided by the mean) gives a more robust indicator of system transition, and this has been successfully applied before in the context of disease elimination (Drake and Hay, 2017). The coefficient of variation is easily calculated using our predictions of the mean and the variance, and represents the understanding that context of the data is important: a variation from the mean is only big or small compared to that mean.

As before we can see that the prediction made using the steady state values (Fig. 2(d) green line) (O’Regan and Drake, 2013) is somewhat different to that using the dynamically changing system (2(d) purple line, Eqs. (1) and (5)). The ‘real’ CV (using the statistics over multiple simulations) for the system going extinct (Ext) follows our prediction very closely (see supplementary figure S1(c)), and this is very different from the CV of the system that doesn’t reach elimination (NExt), and from the system with fixed *β* (FBeta). In particular, the CV does not asymptote exactly at the threshold, but instead a little while after. However, one can clearly see in the CV NExt the point at which progress stalled. As before, detrending using the mean of many simulations produces very good results, as does using the mean of only a few simulations (Fig. 2(d)). However, the CV is more robust to less accurate detrending, and can still produce reasonable results using windowed detrending (see supplementary figure S1(d),(e)). The response is somewhat delayed compared to our predictions, but rises sharply after the threshold while NExt remains flat.

#### 2.3.3 Receiver operating characteristic curves

To determine how predictive our indicators are, we calculate receiver operating characteristic (ROC) curves for our various scenarios. In the literature these are often calculated by comparing indicators for simulations in which *R*_0_ is reduced below 1 with those when *R*_0_ is constant. Since we are considering a scenario in which eradication efforts are underway, but may or may not be successful, we instead will compare with simulations where *R*_0_ is initially decreasing, but does not reduce past 1. For each potential indicator we consider multiple timepoints starting at the time at which $R_{0}=1$ and at each timepoint we calculate a ROC curve in the following way. We take 50 simulations of Ext and 50 simulations of NExt and calculate the indicator for each simulation at the given timepoint. Then we determine whether the indicator is higher (for the CV) or lower (for the variance) than some threshold value at that timepoint and determine whether each simulation is classified correctly by that threshold. This gives a proportion of true positives and false positives, which we can plot as a point on the ROC diagram. So, for example a true positive would be when the CV (variance) of a Ext simulation has exceeded (is lower than) the threshold, whereas a false positive would be when the CV (variance) of a NExt simulation has exceeded (is lower than) the threshold. By changing the threshold value we can move from no positive classifications to all positive. We aim to have the maximum number of true positives and the minimum number of false positives, so the best indicators should lie as close as is possible to the top right corner of the diagram, where we correctly identify all Ext simulations. Note that the intention here is to certify when we believe we have passed the critical transition (and so may cease efforts), rather than to predict when that will happen, and so the earliest timepoint considered is at $R_{0}=1$. For each ROC curve we give the area under the curve (AUC) as an indication of how generally predictive the indicator is.

We find that the variance (Fig. 2(e)) generally performs less well than the CV (Fig. 2(f)), as expected from the previous sections. However, when taking a simple threshold as described, the windowed detrending (see Fig. 2(e) and (f), compared to supplementary figures S2(a)–(d)) method results in the most promising ROC curves, even though the curves are furthest from those predicted (Fig. 2(b)). This is because, although the mean value is predicted accurately, the curves are very noisy when detrending using multiple populations, since the windowed detrending smooths the signal. Thus even though the red and blue curves are closer together in Fig. 2(b), the shaded areas are more separated. It should be noted that in practice there may be better ways to classify the data other than a simple threshold. For example, one might look at the long term trends to see where changes may occur, or to compare these indicators to those in similar places where elimination has been achieved. Another approach that has been suggested in the literature is to use Kendall’s tau to quantify whether an upwards or downwards trend is seen in the indicator and consider whether this value has exceeded a given threshold. Since the CV is continuously increasing in Ext simulations for all detrending methods, and flattens for NExt, this is extremely successful, giving a ROC curve that is almost indistinguishable from the left and top axes. In contrast for the variance, the ROC curves are very close to the x=y line (see supplementary material S2(e), (f)), representing very little predictive power of Kendall’s tau in this case.

#### 2.3.4 Other detrending methods

Other detrending methods can also be considered before calculating potential indicators of elimination for a single population. For all detrending methods that were considered, the resulting CV remained more similar than the variance, supporting our result that the CV is more robust to detrending methods than the variance. Other methods considered (Figures not shown here) are given below

• **Gaussian detrending:** the timeseries is smoothed by windowed average with gaussian weighting, so that data close to the timepoint considered are weighted more highly than those further away. For smaller windows this is very similar to the windowed mean detrending whereas for larger windows spurious oscillations became apparent, particularly close to disease extinction. This method has been used successfully to detrend historical climate records to indicate abrupt climate change shifts (Dakos et al., 2008) and further work has been carried out analysing the sensitivity of the bandwidth used for filtering (Lenton et al., 2012).
• **Windowed linear regression:** linear regression is undertaken on each moving window. This gives similar results to Gaussian detrending, with undesirable excursions away from the prediction, even for moderately sized windows (Lenton et al., 2012).
• **Windowed quadratic regression:** quadratic regression is undertaken on each moving window. This has not been considered in the critical slowing literature, and gives good results at smaller window sizes, getting progressively closer to the predictions for larger windows, however, spurious results still dominate for larger windows before reaching the predicted levels.
• **Wavelets:** wavelets are used to fit the timeseries before discarding higher order wavelets to smooth the signal. This is a large class of methods, corresponding to different wavelets with different levels of smoothing and, in principle, may represent an as-yet under-explored class of techniques for critical slowing down (Dakos et al., 2012). In particular it may be possible to design specific wavelets with this application in mind. However, using more classical wavelets, such as the Daubechies extremal-phase wavelets or symlets, it is very difficult to get robust results close to the predictions, and instead we obtain very noisy signals with a lot of spurious oscillations.

In addition to the potentially under-explored promise of wavelets as a detrending method, they are also often used to directly estimate the (total) variance in a signal (Nason and Von Sachs, 1999). One possible route to estimating time-varying statistics, therefore, would be to attempt to adapt this method to a non-stationary signal.

## 3 Metapopulations

The successful detrending using the mean of even a small number of simulations suggests that detrending using the mean of a number of weakly coupled populations may prove fruitful. In epidemiology this is referred to as a metapopulation model, and we begin by considering the simplest possible version of this model, in which *M*^2^ subpopulations are connected together in a grid. Subpopulations that are next to each other may swap individuals as shown in Table 3. The following methods we used to calculate statistical indicators are widely used in the literature and have previously been used to derive a metapopulation model with constant transmission rates to capture the frequency and amplitude for infection dynamics with stochastic oscillations (Rozhnova, Nunes, McKane, 2012, Rozhnova, Nunes, McKane, 2011).

**Table 3:** Transition probabilities for the metapopulation model, where *d_i_* is the degree of population *i*, and $N_{M}=\frac{N}{M^{2}}$ is the size of each metapopulation.

Event	Change in state	Transition rates
Infection in population *i*	$\left(I_{i}\right)\to \left(I_{i}+1\right)$	$\beta \frac{\left(N_{M}-I_{i}\right)I_{i}}{N_{M}}$
Recovery in population *i*	$\left(I_{i}\right)\to \left(I_{i}-1\right)$	*γI_i_*
Movement between individuals *I_i_* and *S_j_* in adjacent populations	$\left(I_{i},I_{j}\right)\to \left(I_{i}-1,I_{j}+1\right)$	$\rho \frac{I_{i}\left(N_{M}-I_{j}\right)}{N_{M}}$

We calculate statistical indicators similarly to Section 2.1 (see Supplementary Information Section 3), using the linear noise approximation:

$$\frac{I_{i}}{N_{M}}=\phi_{i}\left(t\right)+\frac{1}{\sqrt{N_{M}}}\zeta_{i},$$

obtaining

$$\frac{d\phi_{i}}{dt}=\beta \phi_{i}\left(1-\phi_{i}\right)-\gamma \phi_{i}$$

and the multivariate Fokker–Planck equation for the stochastic variable, *ζ*:

$$\frac{\partial \Pi (\zeta ,t)}{\partial t}=-\sum_{i,j}A_{ij}\frac{\partial }{\partial \zeta_{i}}\left(\zeta_{j}\Pi \right)+\frac{1}{2}\sum_{i,j}B_{ij}\frac{\partial^{2}\Pi }{\partial \zeta_{i}\partial \zeta_{j}},$$

where

(6) $$\begin{array}{ccc} A_{ii} & = & -(\beta \left(2\phi -1\right)+\gamma +d_{i}\rho ), \end{array}$$

(7) $$\begin{array}{ccc} A_{ij} & = & \rho ,\;\text{if}\;i\;\text{and}\;j\;\text{are}\;\text{adjacent,} \end{array}$$

(8) $$\begin{array}{ccc} B_{ii} & = & \left(\beta +2\rho d_{i}\right)\phi \left(1-\phi \right)+\gamma \phi , \end{array}$$

(9) $$\begin{array}{ccc} B_{ij} & = & -2\rho \phi (1-\phi ),\;\text{if}\;i\;\text{and}\;j\;\text{are}\;\text{adjacent,} \end{array}$$

and *d_i_* is the degree of population *i*. Here we use that the mean field solution is the same for all *i* (for identical initial conditions). The solution to the linear FPE is Gaussian and is fully described by the first and second moments. The covariance matrix Θ, where each element equals, $\Theta_{ij}=\langle \langle \zeta_{i}\zeta_{j}\rangle \rangle =\langle \zeta_{i}\zeta_{j}\rangle -\langle \zeta_{i}\rangle \langle \zeta_{j}\rangle$ is given by:

(10) $$\partial_{t}\Theta =A\Theta +\Theta A^{T}+B.$$

This equation may be solved numerically over time, or analytically at steady state. Solving at steady state before substituting in a time-varying value for *β* allows for an analytical solution at the cost of assuming that the system is at steady state for each value of *β*. This becomes progressively less accurate as we approach the critical transition, as the time to reach the steady state increases. For four populations the steady state solution gives:

(11) $$v\left(t\right)=\frac{\gamma \left(2\rho^{2}\left(4\beta -3\gamma \right)+2\rho \left(\gamma -3\beta \right)\left(\gamma -\beta \right)+\beta {\left(\beta -\gamma \right)}^{2}\right)}{\beta^{2}\left(\beta -\gamma +2\rho \right)\left(\beta -\gamma +4\rho \right)}.$$

and for 9 populations this depends on the degree of the node (i.e. how many other populations that population is connected to). Taking a weighted sum across these populations we obtain:

(12) $$\begin{array}{ccc} v(t) & = & \frac{\gamma^{2}\left(\beta \left(t\right)-\gamma \right)}{\beta {\left(t\right)}^{2}}(\frac{2}{\beta (t)-\gamma +\rho }+\frac{1}{\beta (t)-\gamma +2\rho }\\ & & +\;\frac{2}{\beta (t)-\gamma +3\rho }+\frac{2}{\beta (t)-\gamma +4\rho }+\frac{1}{\beta (t)-\gamma +6\rho })\\ & & +\frac{9\gamma }{\beta (t)}-\frac{8\gamma^{2}}{\beta {\left(t\right)}^{2}}. \end{array}$$

### 3.1 Spatially detrending simulations

From the success of simulation detrending using only four realisations of exactly the same system, we investigated detrending simulations spatially by subtracting the mean infectious behaviour over multiple connected subpopulations (over 4 and 9 subpopulations, given in Fig. 3). We propose this detrending method with the aim that it is applicable with real-world epidemiological data that has been collected spatially, where one might observe disease elimination in neighbouring subpopulations. Fig. 1(b) presents a graphical figure demonstrating how we calculated the indicators with spatial detrending. This calculation is comparable with the “spatial variance” statistic that has been explored previously for ecological systems (Kéfi, Guttal, Brock, Carpenter, Ellison, Livina, Seekell, Scheffer, Van Nes, Dakos, 2014, Guttal, Jayaprakash, 2009). However, it should be noted that spatial detrending uses the metapopulation framework to remove the mean behaviour such that many other potential indicators could be implemented on the detrended timeseries, not just the variance.

### 3.2 Results for the metapopulation model

We wish to compare our two analytical predictions (varying and at steady state) with simulated data. From these data we may calculate three different statistics (summarised in Fig. 1 (b)), and discussed in more detail below. In the following we use the same total population size as before (N=20,000) and divide this total population into *M*^2^ subpopulations. In each figure, the purple line is the analytical prediction obtained by numerically solving Eq. (10); the green line is the steady state variance obtained by solving Eqs. (11) and (12); the red line is the simulation when infection begins to increase rather than go extinct at $R_{0}=1$ (NExt); the blue line is the simulation when infection goes to elimination (Ext).

#### 3.2.1 Variance

Firstly, the variance at each timepoint calculated over multiple stochastic simulations of the model was calculated for NExt and Ext simulations (Fig. 3(a)). This corresponds to the quantity we derive analytically in Eq. (10) but, as before, is not calculable with real data since it requires multiple realisations of the same process. Secondly, we find the variance between the different subpopulations. This may approximate the true variance if the subpopulations are not strongly coupled (*i.e.* if *ρ* is not too large). However for a single realisation with only a few subpopulations, this is very noisy. The third quantity therefore is the moving window time average of the variance between the different subpopulations (Fig. 3(b) and (c)), which provides a less noisy signal for small numbers of subpopulations.

Our analytical prediction compares well for both four and nine subpopulations when calculating the variance over multiple realisations (Fig. 3 (a) and Supplementary Figure S3(a)). This demonstrates that the linear noise approximation has worked well in this system. For four subpopulations we already find significantly better results by detrending using the mean of the four subpopulations (Fig. 3(b)) than when using windowed detrending for a single population (Fig. 2(b)). Fig. 3(b) also demonstrates that taking the moving-window average of the variance successfully reduces the noise while maintaining a good correspondence to the analytical prediction. However there is still an appreciable difference between the prediction and simulated statistics.

By moving to nine subpopulations (Fig. 3(c)) we see a substantial increase in both the predicted signal (*i.e.* the variance increases more as we approach the threshold) and the correspondence between the simulated statistics and the data. This is apparent even when only one simulation is used, when the signal is smoothed using a windowed average (supplementary information Figure S3(b),(c)). Increasing the number of subpopulations further to M=5 (*i.e.* 25 subpopulations), leads to even more accurate predictions (result not shown). We expect that increasing *ρ* leads to stronger coupling between the subpopulations and hence taking the average of the populations may be a less effective detrending method. Sensitivity analysis (supplementary information Figure S4) with varying values of *ρ* and the initial value of *R*_0_ (so that the change in *β* over time is quicker) shows that in general larger values of *ρ* does lead to larger errors, while the sensitivity to *R*_0_ is more subtle.

#### 3.2.2 Coefficient of variation

As before, we wish to also compare the analytical predictions for coefficient of variation with simulated data. In Fig. 3(d) we calculate potential indicator CV by detrending the simulated data using the mean of 4 subpopulations and then we calculate the coefficient of variation on a moving average of 50 timepoints. This figure was calculated using only a single set of time series data (e.g. collected data from *M*^2^ neighbouring subpopulations) with the mean (solid line) taken over all the subpopulations and one standard deviation about that mean (shaded region). With only a single realisation, we can produce very good results which are comparable to the behaviour of the analytical solution, even when only using 4 subpopulations. Detrending and calculating the statistic over more subpopulations, i.e. when $M^{2}=9,$ smooths the signal and improves the correspondence between the simulated statistics and analytical solution (see supplementary Figure S5(e)). We have included (supplementary information Figure S5) results from calculating the CV over 50 realisations by detrending: between realisations and between subpopulation. These results are comparable to those shown here and closely follow the analytical solutions.

#### 3.2.3 ROC Curves

We evaluated receiver operating characteristic curves for 4 and 9 metapopulations, following a similar analysis of early warning signals (coefficient of variation and variance) of the SIS model in Section 2.3.3. A linear threshold of the EWS was used to find multiple ROC curves at various time points (t=400 to t=450). We classify the simulations NExt and Ext at the threshold to get the true positive and false positive rates. Similarly to the single population model, variance generally performs less well than the CV. However, it is surprising that detrending by using the mean infectives over all subpopulations (i.e. a method that is achievable with only one realisation, Fig. 3(e)) performs better (AUC measure is closer to 1) than detrending over 50 realisations (Fig. 3(f)). As for a single population, using the Kendall-tau coefficient is less predictive than a simple threshold, and detrending by subpopulation performs slightly better than detrending over 50 realisations (supplementary Figure S6 (variance) and S7 (CV)).

## 4 Discussion

Our results show that detrending is the main cause of lack of correspondence between analytical predictions and simulated statistics, and not the adoption of moving window statistics nor the approximation of the discrete system by a continuous variable. Critical slowing down theory uses the fact that a dynamical system undergoing a bifurcation in which the stability of the steady state changes must therefore have eigenvalues passing through zero and thus the relaxation time to the steady state must increase. The principle of using critical slowing down theory to determine indicators of system transition in the absence of a specific model relies upon the calculated indicators consistently representing that increase in relaxation time. Thus it is necessary to determine how to process data in a way that preserves the relationship to the ‘true’ statistics of the stochastic system.

We demonstrate here that detrending in metapopulation models using the mean of all subpopulations shows promise as a means of analysing spatially distributed systems. Even when using only four or nine subpopulations, the simulated results fit the predictions much better than when using windowed detrending in a single population. In spite of this, we find that windowed detrending can result in a more predictive indicator of elimination both in a single population and in a metapopulation. This is not a contradiction, since our analytical predictions show that the indicators studied increase close to the transition, but not always quickly or exactly at the transition. Although the relaxation time to the steady state demonstrates a sharp transition at the bifurcation point, this is primarily seen in the statistics directly about that steady state, and is not seen as clearly in a slowly temporally varying system. This is to be expected, as by the theory itself we expect the system to stay further from the true steady state for longer as we approach the transition.

It is interesting to compare detrending using the mean of multiple subpopulations with a seasonal system, in which one might detrend using historic data. The question of seasonal infection data has been studied by Miller et al. (2017), who found that removing seasonal trends did not improve early warning indicators uniformly. In contrast to our results, Miller et al. concluded that seasonal detrending is often not advantageous. However, the authors did not make analytical predictions of their indicators and did not show whether their simulated statistics would correspond to the ‘true’ statistics when calculated over many realisations. It is possible that although seasonal detrending did not improve the predictiveness of their indicators, it may still have resulted in statistics that are closer to those of the stochastic system and thus are more easily understood and studied. In addition, while Miller et al. studied outbreak dynamics, we are interested in indicators of eradication, in which historical dynamics may be more useful to determine the general trend.

The problem of good detrending is not a new one, and has been discussed in the past, particularly with regards to early warning signs in climate change. Drake et al. remark that good detrending is essential, since residual trends in the data will produce spurious signals, particularly in the autocorrelation (Dakos et al., 2008). The effects of various detrending methods and moving window sizes is also studied by Lenton et al., who consider linear detrending and Gaussian filtering, as well as detrended fluctuation analysis (DFA) (Lenton et al., 2012). Both linear detrending and Gaussian filtering fit a function to the dataset: either a linear function on multiple smaller windows; or a Gaussian kernel smoothing function across the whole timeseries. The authors determine how successful each method is by applying it to real data in which a transition occurs at a known point and looking for indicators at this point and not before. While linear detrending is sometimes found to be insufficient, Gaussian filtering or DFA often give the desired results. The use of real data in this case is both encouraging and challenging. It is important to be able to show that the theory can work on real data and will give consistent indicators of known transitions. However it is also difficult to know what result we are expecting to see, since there is no ‘true’ statistic to compare to. We therefore favour a dual approach: understanding and analysing our indicators on model systems first, ensuring that our predictions are robust to the detrending method used, before applying them to real world data in the future.

## 5 Conclusions

As we strive to reach the 2020 goals for the elimination of NTDs, the question of how to ensure a disease has reached the threshold for elimination is a timely and important one. We present here a statistical tool that can give evidence for whether we have reached that goal. It is important to be able to move resources elsewhere once the threshold has been reached. However, if elimination efforts cease too quickly we risk disease resurgence, negating all the hard work to bring prevalences down. It is therefore imperative to be risk-averse in this decision, and err on the side of fewer ‘false positives’ when classifying if the threshold has been reached.

It is clear from our analysis that it is the detrending of the signal that is the single biggest barrier to our analysis of potential indicators of threshold changes. While badly detrended data can still be highly predictive for some indicators (e.g. CV), the difference from our predictions makes it hard to understand when this will be the case. In addition, the underlying theory of critical slowing down only really applies when considering a properly detrended signal, as can be seen by our lack of success with badly detrended simulations. If a more reliable method can be found this may be more generally applicable without the need to understand each individuals system in detail. More work is therefore needed to understand how the detrending method interacts with the predicted behaviour of the indicators and to develop better detrending methods that do not distort the statistical properties of the noise.

For epidemiological applications we have demonstrated that using multiple subpopulations in a metapopulation model allows for much better detrending and correspondingly better statistical indicators of a critical transition. This is particularly useful for models of endemic diseases, and also gives hope for the use of spatial data. More work is required, however, to extend these analyses to more realistic models of current diseases. Firstly, our assumption that all the metapopulations have identical parameters is clearly unrealistic, and work needs to be done to relax that assumption. While the SIR model and models of vector-borne diseases have been studied in the single population framework (O’Regan, Drake, 2013, O’Regan, Lillie, Drake, 2015), we would like to extend these analyses to our metapopulation model. In addition, future work will focus on extending our analysis to more realistic models of specific diseases. Finally, current disease eradication campaigns, particularly in NTD communities, often focus on mass drug administration, in which the community is repeatedly treated at regular intervals to reduce prevalence, which has not been studied under the critical slowing down framework.

## Appendix A Supplementary materials

Supplementary Data S1 Supplementary Raw Research Data. This is open data under the CC BY license http://creativecommons.org/licenses/by/4.0/ Supplementary Data S1
