# Supplementary material for: The problem of detrending when analysing potential indicators of disease elimination
Source: J Theor Biol. 2019 Nov 21;481:183–93. doi: 10.1016/j.jtbi.2019.04.011 (PMC6859505; doi:10.1016/j.jtbi.2019.04.011)
Supplement: Supplementary Data S1 — Supplementary Raw Research Data. This is open data under the CC BY license http://creativecommons.org/licenses/by/4.0/ [file mmc1.pdf]

Supplementary Information: The problem of detrending when analysing potential indicators of disease elimination

1 Deviation of the Fokker-Planck equation for SIS dynamics:

For the SIS model without metapopulations, the mean-field equations are given by $\frac{d\phi}{dt} = \beta\phi(1-\phi) - \gamma\phi$ where $\phi = \frac{\langle I \rangle}{N}$.

The following deviation follows from van Kampen (Chapter 8 and 10). The linear noise approximation for the discrete infectious state I is given by:

$$I = N\phi(t) + N^{1/2}\zeta. \tag{1}$$

The general form of the master equation for the SIS model based on the transition probabilities given in Table 1 is,

$$\begin{aligned}\frac{dP(I,t)}{dt} &= T(I|I-1)P(I-1,t) + T(I|I+1)P(I+1,t) - T(I-1|I)P(I,t) - T(I+1|I)P(I,t) \\ &= \frac{\beta(t)\,(N-(I-1)(I-1))}{N}P(I-1,t) + \gamma(I+1)P(I+1,t) - \gamma IP(I,t) - \frac{\beta(t)(N-I)I}{N}P(I,t)\end{aligned} \tag{2}$$

The master equation can be written using step operators which act on an arbitrary function of n, defined as $\mathbb{E}f(n) = f(n+1)$ and $\mathbb{E}^{-1}f(n) = f(n-1)$.

$$\begin{aligned}\frac{dP(I,t)}{dt} &= \mathbb{E}^{-1}T(I+1|I)P(I,t) + \mathbb{E}T(I-1|I)P(I,t) - T(I-1|I)P(I,t) - T(I+1|I)P(I,t) \\ &= (\mathbb{E}^{-1}-1)T(I+1|I)P(I,t) + (\mathbb{E}-1)T(I-1|I)P(I,t) \\ &= (\mathbb{E}^{-1}-1)\frac{\beta(t)(N-I)I}{N}P(I,t) + (\mathbb{E}-1)\gamma IP(I,t) \qquad (3) \\ P(I,0) &= \delta_{I,I_0}.\end{aligned}$$

The step operators have a simple expansion involving powers of $N^{-1/2}\partial/\partial\zeta$. Since the operators take I to $I+1$ then it follows that it takes, $\zeta = \frac{I-N\phi(t)}{N^{1/2}}$ to$\frac{I+1-N\phi(t)}{N^{1/2}} = \zeta + \frac{1}{N^{1/2}}$. From here, we can perform a Taylor expansion and derive the following expression for $\mathbb{E}$:

$$\begin{aligned}\mathbb{E}f(\zeta) &= f(\zeta + N^{-1/2}) \\ &= f(\zeta) + N^{-1/2}f'(\zeta) + \frac{1}{2}(N^{-1/2})^2 f''(\zeta) + ... \\ \mathbb{E} &= 1 + N^{-1/2}\frac{\partial}{\partial\zeta} + \frac{1}{2}N^{-1}\frac{\partial^2}{\partial\zeta^2} + ... \\ \mathbb{E}^{-1} &= 1 - N^{-1/2}\frac{\partial}{\partial\zeta} + \frac{1}{2}N^{-1}\frac{\partial^2}{\partial\zeta^2} - ...\end{aligned}$$

Define a new probability distribution function Π by $P(I,t) = \Pi(\zeta,t)$. The derivative of the probability distribution function with respect to t,

$$\frac{\partial P(I,t)}{\partial t} = \frac{\partial \Pi}{\partial \zeta}\frac{d\zeta}{dt} + \frac{\partial \Pi}{\partial t} = -N^{1/2}\frac{d\phi}{dt}\frac{\partial \Pi}{\partial \zeta} + \frac{\partial \Pi}{\partial t} \tag{4}$$

is needed for deriving the continuous space master equation.

Combining equations 3 and 4 together, we can write down the continuous space master equation:

$$\begin{aligned}
-N^{1/2}\frac{d\phi}{dt}\frac{\partial \Pi}{\partial \zeta} + \frac{\partial \Pi}{\partial t} &= (\mathbb{E}^{-1} - 1)T(I+1|I)P(I,t) + (\mathbb{E} - 1)T(I-1|I)P(I,t) \\
&= (-N^{-1/2}\frac{\partial}{\partial \zeta} + \frac{1}{2}N^{-1}\frac{\partial^2}{\partial \zeta^2} - ...)T(I+1|I)\Pi(\zeta,t) \\
&+ (N^{-1/2}\frac{\partial}{\partial \zeta} + \frac{1}{2}N^{-1}\frac{\partial^2}{\partial \zeta^2} + ...)T(I-1|I)\Pi(\zeta,t) \\
&\quad \text{and substitute the linear approximation} \\
&\approx (-N^{1/2}\frac{\partial}{\partial \zeta} + \frac{1}{2}\frac{\partial^2}{\partial \zeta^2})\beta(1 - \phi - N^{-1/2}\zeta)(\phi + N^{-1/2}\zeta)\Pi(\zeta,t) \\
&+ (N^{1/2}\frac{\partial}{\partial \zeta} + \frac{1}{2}\frac{\partial^2}{\partial \zeta^2})\gamma(\phi + N^{-1/2}\zeta)\Pi(\zeta,t).
\end{aligned} \tag{5}$$

We collect powers of N in equation 5 and substitute the mean-field deterministic approximation as $N \to \infty$ (macroscopic description which ignores fluctuations). This results in the linear Fokker-Planck equation for this system:

$$\begin{aligned}
\frac{\partial \Pi}{\partial t} &= N^{1/2}(\beta\phi(1-\phi) - \gamma\phi)\frac{\partial \Pi}{\partial \zeta} + (N^{1/2}\frac{\partial}{\partial \zeta} + \frac{1}{2}\frac{\partial^2}{\partial \zeta^2} + ...)\gamma(\phi + N^{-1/2}\zeta)\Pi(\zeta,t) \\
&+ (-N^{1/2}\frac{\partial}{\partial \zeta} + \frac{1}{2}\frac{\partial^2}{\partial \zeta^2} - ...)(\beta\phi(1-\phi) + \beta(1-2\phi)N^{-1/2}\zeta - \beta\phi N^{-1/2}\zeta)\Pi(\zeta,t) \\
&\quad \text{we collect terms of order } N^0, \\
\frac{\partial \Pi}{\partial t} &= -(\beta - 2\beta\phi - \gamma)\frac{\partial \zeta\Pi}{\partial \zeta} + \frac{1}{2}(\beta(1-\phi)\phi + \gamma\phi)\frac{\partial^2 \Pi}{\partial \zeta^2}.
\end{aligned}$$

The solution for the analytical variance can be deduced from the following equations,

$$\frac{\partial \langle \zeta \rangle_t}{\partial t} = (\beta - \gamma - 2\beta\phi)\langle \zeta \rangle_t, \tag{6}$$

$$\begin{aligned}
\frac{\partial \langle \zeta^2 \rangle_t}{\partial t} &= 2(\beta - \gamma - 2\beta\phi)\langle \zeta^2 \rangle_t + \beta(1-\phi)\phi + \gamma\phi \\
&= N\frac{dV}{dt} = (\beta - \gamma - 2\beta\phi)NV + \beta(1-\phi)\phi + \gamma\phi.
\end{aligned} \tag{7}$$

At steady state when $\frac{d\phi}{dt} = 0$ and $\frac{dV}{dt} = 0$, we obtain $\phi* = 1 - \frac{\gamma}{\beta}$ and $V* = \frac{1}{N}\frac{1}{R_0}$

2 Single Population Model

2.1 Potential Indicators: variance and coefficient of variation

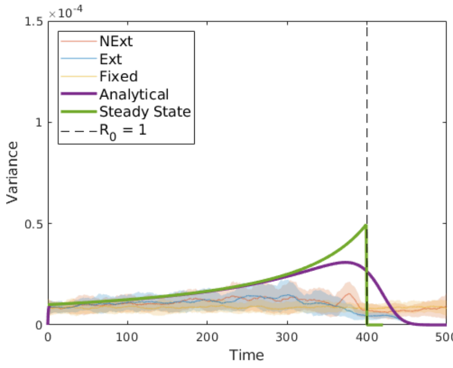

(a) Variance, windowed detrending using 200 timepoints

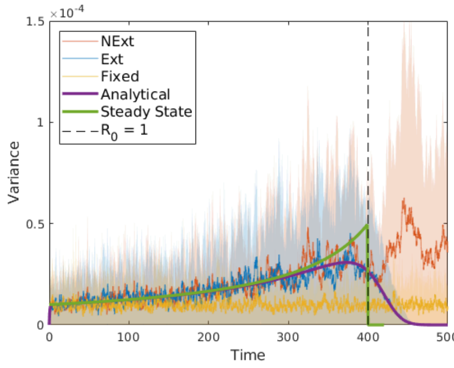

(b) Variance, detrending using 50 realisations

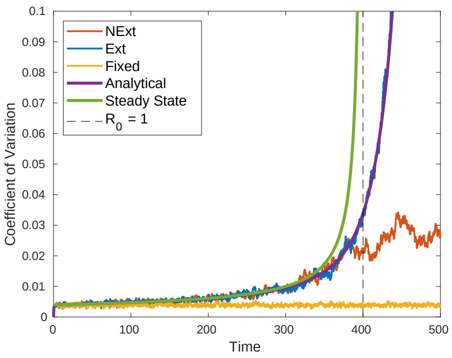

(c) CV over 50 realisations

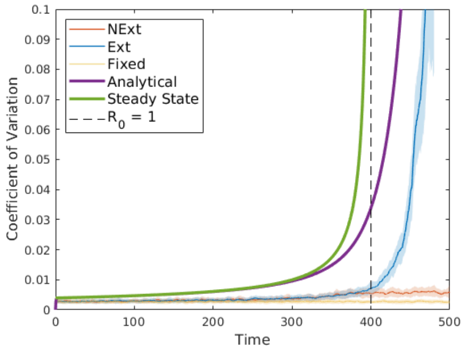

(d) CV, windowed detrending using 50 timepoints

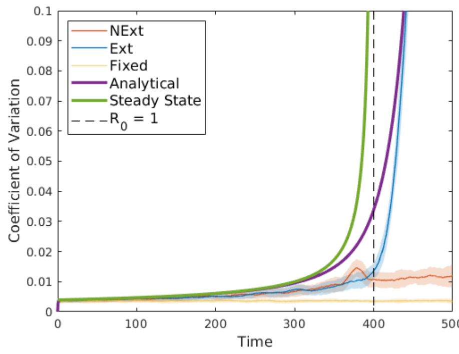

(e) CV, windowed detrending using 200 timepoints

Figure 1: **Single population**: comparing predictions to simulations for: (a) variance, over a moving window of size 200 timepoints; (b) variance, over a moving window of size 50 timepoints after detrending using 50 simulations; (c) CV, over 50 realisations; (d) CV, over a moving window of size 50 timepoints; and (e) CV, over a moving window of size 200 timepoints. Each figure shows: steady state predictions (green line); dynamic predictions (purple line); simulations of the model going extinct (Ext, blue line); simulations of the model not going extinct (NExt, red line); and simulations of the model with fixed β (FBeta, yellow line). For repeated simulations each line is the mean value obtained over 50 simulations and the shaded area represents one standard deviation about the mean.

2.2 ROC curve analysis

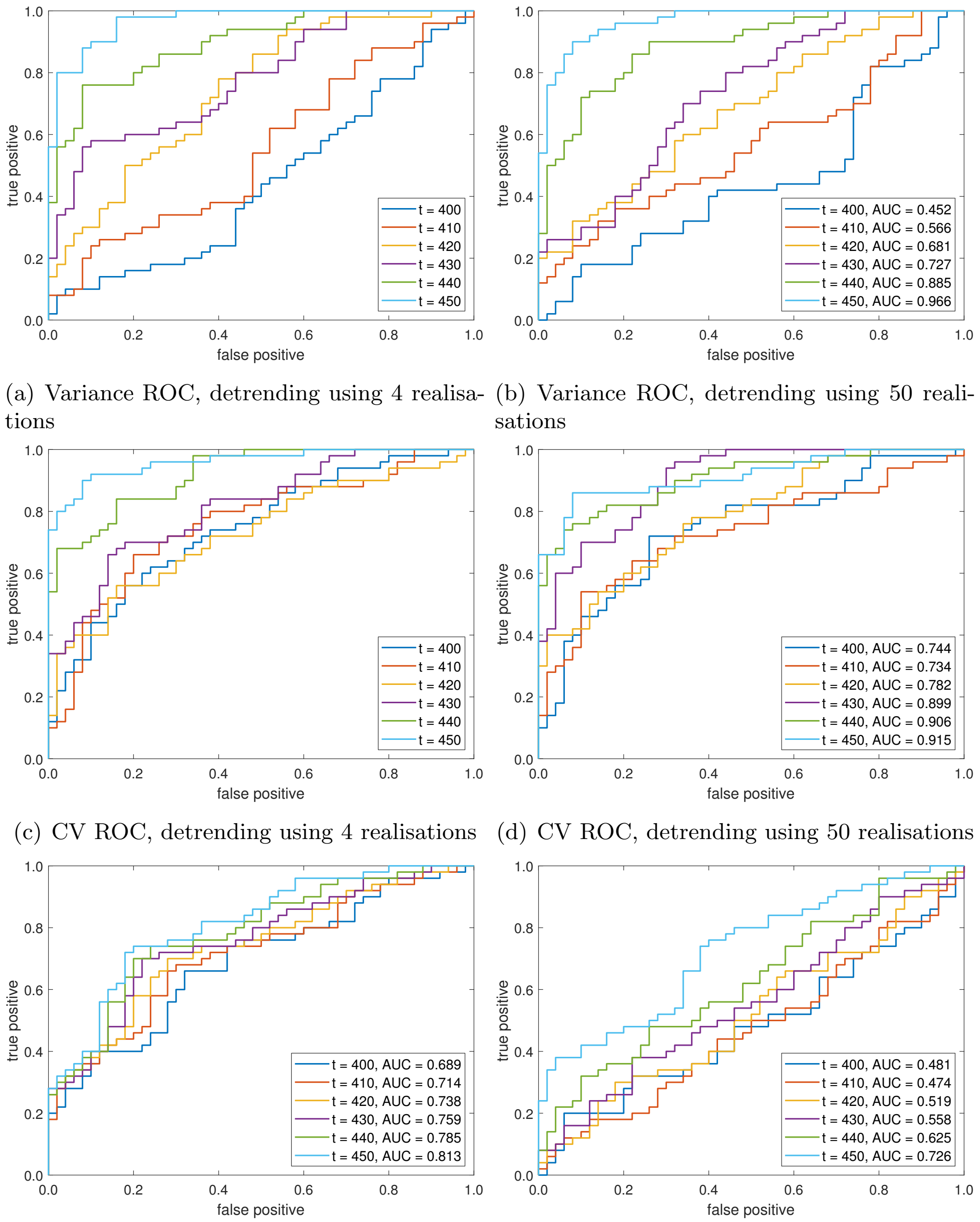

(a) Variance ROC, detrending using 4 realisations

(b) Variance ROC, detrending using 50 realisations

(c) CV ROC, detrending using 4 realisations

(d) CV ROC, detrending using 50 realisations

(e) Variance Kendall-tau, windowed detrending using 200 timepoints

(f) Variance Kendall-tau, detrending using 50 realisations

Figure 2: **Single population**: ROC curves calculated over 50 realisations at various timepoints by thresholding in variance ((a), (b)); thresholding in CV ((c), (d)); or using Kendall's tau ((e) and (f)). Each curve calculates the statistic on a moving window of size 50 timepoints after detrending using: (a) and (c) mean values over 4 realisations; (b), (d) and (f) mean values over 50 realisations; and (e) windowed detrending with a window of size 200 timepoints. Each ROC curve the legend gives the area under the curve (AUC), suggesting how predictive that indicator is (AUC closer to 1 are more predictive).

3 Deviation of the Fokker-Planck equation for metapopulation dynamics:

3.1 Derivation for 4 populations

We write the transition probabilities as given in Table 3 in terms of the step operators. For example, the transition probability of an susceptible individual in population 1 becoming infected can be written as $T(I_1, ...|I_1-1, ...) = \mathbb{E}_{I_1}T(I_1+1, ...|I_1)$. The total population size was taken to be $20,000$ and we divide this equally (depending on the number of subpopulations M^2) assuming that the size of each subpopulation, N_M, is the same. The master equation for $M^2 = 4$ subpopulations on a lattice, $P(I_1, I_2, I_3, I_4, t)$, is the probability observing $\mathbf{I}$ infectives at time t,

$$
\begin{aligned}
\frac{dP(I_1,I_2,I_3,I_4,t)}{dt} &= (\mathbb{E}_{I_1}^{-1}-1)T(I_1+1,...|I_1,...)P(I_1,...,t) + (\mathbb{E}_{I_2}^{-1}-1)T(I_2+1|I_2)P(...,t) \\
&+ (\mathbb{E}_{I_3}^{-1}-1)T(I_3+1|I_3)P(...,t) + (\mathbb{E}_{I_4}^{-1}-1)T(I_4+1|I_4)P(...,t) \\
&+ (\mathbb{E}_{I_1}-1)T(I_1-1,...|I_1,...)P(I_1,...,t) + (\mathbb{E}_{I_2}-1)T(I_2-1|I_2)P(...,t) \\
&+ (\mathbb{E}_{I_3}-1)T(I_3-1|I_3)P(...,t) + (\mathbb{E}_{I_4}-1)T(I_4-1|I_4)P(...,t) \\
&+ (\mathbb{E}_{I_1}^{-1}\mathbb{E}_{I_2}-1)T(I_1+1,I_2-1,...|I_1,I_2,...)P(I_1,I_2,..,t) \\
&+ (\mathbb{E}_{I_2}^{-1}\mathbb{E}_{I_1}-1)T(I_1-1,I_2+1|I_1,I_2)P + (\mathbb{E}_{I_3}^{-1}\mathbb{E}_{I_1}-1)T(I_1-1,I_3+1|I_1,I_3)P \\
&+ (\mathbb{E}_{I_1}^{-1}\mathbb{E}_{I_3}-1)T(I_1+1,I_3-1|I_1,I_3)P + (\mathbb{E}_{I_4}^{-1}\mathbb{E}_{I_2}-1)T(I_2-1,I_4+1|I_2,I_4)P \\
&+ (\mathbb{E}_{I_2}^{-1}\mathbb{E}_{I_4}-1)T(I_2+1,I_4-1|I_2,I_4)P + (\mathbb{E}_{I_4}^{-1}\mathbb{E}_{I_3}-1)T(I_3-1,I_4+1|I_3,I_4)P \\
&+ (\mathbb{E}_{I_3}^{-1}\mathbb{E}_{I_4}-1)T(I_3+1,I_4-1|I_3,I_4)P \\
&= \sum_{i=1}^{4}\Big((\mathbb{E}_{I_i}^{-1}-1)T(I_i+1,...|I_i,...)P(...,t) + (\mathbb{E}_{I_i}-1)T(I_i-1,...|I_1,...)P(...,t)\Big) \\
&+ \sum_{i=1}^{4}\sum_{j\in\mathcal{N}_i}(\mathbb{E}_{I_i}^{-1}\mathbb{E}_{I_j}-1)T(I_i+1,I_j-1|I_i,I_j)P(...,t) \\
\frac{dP(I_1,I_2,I_3,I_4,t)}{dt} &= \sum_{i=1}^{4}\left((\mathbb{E}_{I_i}^{-1}-1)\beta\frac{(N_M-I_i)I_i}{N_M}P(...,t) + (\mathbb{E}_{I_i}-1)\gamma I_i P(...,t)\right) \\
&+ \sum_{i=1}^{4}\sum_{j\in\mathcal{N}_{\mathcal{M}i}}(\mathbb{E}_{I_i}^{-1}\mathbb{E}_{I_j}-1)\rho\frac{(N_M-I_i)I_j}{N_M}P(...,t) \qquad (8)
\end{aligned}
$$

The step operators are defined for each subpopulation i, depending on the linear noise approximation: $I_i = N_M\phi_i + N_M^{1/2}\zeta_i$,

$$
\begin{aligned}
\mathbb{E}_i &= 1 + N_M^{-1/2}\frac{\partial}{\partial\zeta_i} + \frac{1}{2}N_M^{-1}\frac{\partial^2}{\partial\zeta_i^2} + ... \\
\mathbb{E}_i^{-1} &= 1 - N_M^{-1/2}\frac{\partial}{\partial\zeta_i} + \frac{1}{2}N_M^{-1}\frac{\partial^2}{\partial\zeta_i^2} + ... \\
\mathbb{E}_i\mathbb{E}_j^{-1} - 1 &= N_M^{-1/2}\left(\frac{\partial}{\partial\zeta_i} - \frac{\partial}{\partial\zeta_j}\right) + \frac{N_M^{-1}}{2}\left(\frac{\partial}{\partial\zeta_i} - \frac{\partial}{\partial\zeta_j}\right)^2 + ...
\end{aligned}
$$

Similarly to the simple SIS model, we define a new probability distribution function Π by $P(I_1, I_2, I_3, I_4, t) =$

$\Pi(\zeta_1, \zeta_2, \zeta_3, \zeta_4, t)$. The derivative of the probability distribution function with respect to t,

$$\begin{aligned}
\frac{\partial P(I_1, I_2, I_3, I_4, t)}{\partial t} &= \frac{\partial \Pi}{\partial \zeta_1}\frac{d\zeta_1}{dt} + \frac{\partial \Pi}{\partial \zeta_2}\frac{d\zeta_2}{dt} + \frac{\partial \Pi}{\partial \zeta_3}\frac{d\zeta_3}{dt} + \frac{\partial \Pi}{\partial \zeta_4}\frac{d\zeta_4}{dt} + \frac{\partial \Pi}{\partial t} \\
&= -N^{1/2}\frac{d\phi_1}{dt}\frac{\partial \Pi}{\partial \zeta_1} - N^{1/2}\frac{d\phi_2}{dt}\frac{\partial \Pi}{\partial \zeta_2} - N^{1/2}\frac{d\phi_3}{dt}\frac{\partial \Pi}{\partial \zeta_3} - N^{1/2}\frac{d\phi_4}{dt}\frac{\partial \Pi}{\partial \zeta_4} + \frac{\partial \Pi}{\partial t}
\end{aligned}$$

Substitute the master equation (Equation 8) and step operators into the above equation. Evaluating $\frac{\partial \Pi}{\partial t}$ in the limit $N_M \to \infty$,

$$\begin{aligned}
\frac{\partial \Pi}{\partial t} &= \overbrace{\left[\beta\zeta_1(1-2\rho)\phi_1 + \gamma\zeta_1\right]\frac{\partial}{\partial \zeta_1}\Pi + \rho\left[2\zeta_1 - (\zeta_2+\zeta_3)\right]\frac{\partial}{\partial \zeta_1}\Pi}^{\text{Derivative with respect to: } \zeta_1} \\
&+ \frac{1}{2}\left[\beta(1-\phi_1)\phi_1 + \gamma + \rho(2\phi_1 + \phi_2 + \phi_3 - 2\phi_1\phi_2 - 2\phi_1\phi_3)\right]\frac{\partial^2}{\partial \zeta_1^2}\Pi \\
&+ \overbrace{\left[\beta\zeta_2(1-2\rho)\phi_2 + \gamma\zeta_2\right]\frac{\partial}{\partial \zeta_2}\Pi + \rho\left[2\zeta_2 - (\zeta_1+\zeta_4)\right]\frac{\partial}{\partial \zeta_2}\Pi}^{\text{Derivative with respect to: } \zeta_2} \\
&+ \frac{1}{2}\left[\beta(1-\phi_2)\phi_2 + \gamma + \rho(2\phi_2 + \phi_1 + \phi_4 - 2\phi_2\phi_1 - 2\phi_2\phi_4)\right]\frac{\partial^2}{\partial \zeta_2^2}\Pi \\
&+ \overbrace{\left[\beta\zeta_3(1-2\rho)\phi_3 + \gamma\zeta_3\right]\frac{\partial}{\partial \zeta_3}\Pi + \rho\left[2\zeta_3 - (\zeta_1+\zeta_4)\right]\frac{\partial}{\partial \zeta_3}\Pi}^{\text{Derivative with respect to: } \zeta_3} \\
&+ \frac{1}{2}\left[\beta(1-\phi_3)\phi_3 + \gamma + \rho(2\phi_3 + \phi_1 + \phi_4 - 2\phi_3\phi_1 - 2\phi_3\phi_4)\right]\frac{\partial^2}{\partial \zeta_2^3}\Pi \\
&+ \overbrace{\left[\beta\zeta_4(1-2\rho)\phi_4 + \gamma\zeta_4\right]\frac{\partial}{\partial \zeta_4}\Pi + \rho\left[2\zeta_4 - (\zeta_2+\zeta_3)\right]\frac{\partial}{\partial \zeta_4}\Pi}^{\text{Derivative with respect to: } \zeta_4} \\
&+ \frac{1}{2}\left[\beta(1-\phi_4)\phi_4 + \gamma + \rho(2\phi_4 + \phi_2 + \phi_3 - 2\phi_4\phi_2 - 2\phi_4\phi_3)\right]\frac{\partial^2}{\partial \zeta_4^2}\Pi \\
&- \overbrace{\rho(\phi_1 + \phi_2 - 2\phi_1\phi_2)\frac{\partial^2}{\partial \zeta_1 \partial \zeta_2}\Pi - \rho(\phi_1 + \phi_3 - 2\phi_1\phi_3)\frac{\partial^2}{\partial \zeta_1 \partial \zeta_3}\Pi}^{\text{Covariance terms}} \\
&- \rho(\phi_2 + \phi_4 - 2\phi_2\phi_4)\frac{\partial^2}{\partial \zeta_2 \partial \zeta_4}\Pi - \rho(\phi_3 + \phi_4 - 2\phi_3\phi_4)\frac{\partial^2}{\partial \zeta_3 \partial \zeta_4}\Pi
\end{aligned} \tag{9}$$

The multivariate Fokker-Planck Equation is fully described in terms of matrices A and B, where B is symmetric and positive definite. If both A and B are constant matrices then the solution is Gaussian (linear Fokker-Planck Equation),

$$\frac{\partial \Pi(\zeta, t)}{\partial t} = -\sum_{i,j} A_{ij}\frac{\partial}{\partial \zeta_i}(\zeta_j \Pi) + \frac{1}{2}\sum_{i,j} B_{ij}\frac{\partial^2 \Pi}{\partial \zeta_i \partial \zeta_j}. \tag{10}$$

We assume that the mean-field solution is the same for all populations, $\phi_i = \phi$. Collecting

terms in Equation 9 into the format of the FPE (Equation 10), we arrive at:

$$\begin{aligned}
A_{ii} &= -(\beta(2\phi-1)+\gamma+2\rho), \\
A_{ij} &= \rho, \text{ if } i \text{ and } j \text{ are adjacent}, \\
B_{ii} &= (\beta+2\rho 2)\phi(1-\phi)+\gamma\phi, \\
B_{ij} &= -2\rho\phi(1-\phi), \text{ if } i \text{ and } j \text{ are adjacent},
\end{aligned}$$

3.2 Derivation for general M^2 subpopulations

The general master equation for M^2 subpopulations, $P(\{I_i, i \in [1, M^2]\}, t)$, is the probability observing $\mathbf{I}$ infectives at time t and depends on the number of neighbours, $\mathcal{N}_i$, of each subpopulation i where the degree of each subpopulation i (total number of neighbours) is given by d_i,

$$\begin{aligned}
\frac{dP(\{I_i, i \in [1, M^2]\})}{dt} &= \sum_{i=1}^{M^2} \Big((\mathbb{E}_{I_i}^{-1} - 1) T(I_i + 1, ...|I_i, ...) P(..., t) + (\mathbb{E}_{I_i} - 1) T(I_i - 1, ...|I_1, ...) P(..., t) \Big) \\
&+ \sum_{i=1}^{M^2} \sum_{j \in \mathcal{N}_i} (\mathbb{E}_{I_i}^{-1} \mathbb{E}_{I_j} - 1) T(I_i + 1, I_j - 1 | I_i, I_j) P(..., t) \\
&= \sum_{i=1}^{M^2} \left((\mathbb{E}_{I_i}^{-1} - 1) \beta \frac{(N_M - I_i) I_i}{N_M} P(..., t) + (\mathbb{E}_{I_i} - 1) \gamma I_i P(..., t) \right) \\
&+ \sum_{i=1}^{M^2} \sum_{j \in \mathcal{N}_{\mathcal{M}i}} (\mathbb{E}_{I_i}^{-1} \mathbb{E}_{I_j} - 1) \rho \frac{(N_M - I_i) I_j}{N_M} P(..., t)
\end{aligned}$$

Define a new probability distribution function Π by $P(\{I_i, i \in [1, M^2]\}, t) = \Pi(\{\zeta_i, i \in [1, M^2]\}, t)$. The derivative of the probability distribution function with respect to t,

$$\frac{\partial P(\{I_i, i \in [1, M^2]\}, t)}{\partial t} = \sum_{i=1}^{M^2} \frac{\partial \Pi}{\partial \zeta_i} \frac{d\zeta_i}{dt} + \frac{\partial \Pi}{\partial t} = \sum_{i=1}^{M^2} -N^{1/2} \frac{d\phi_i}{dt} \frac{\partial \Pi}{\partial \zeta_i} + \frac{\partial \Pi}{\partial t}$$

Then following a similar analysis as above (when $M^2 = 4$),

$$\begin{aligned}
\frac{\partial \Pi}{\partial t} &= \sum_{i=1}^{M^2} \left([\beta(1-2\rho)\phi_i + \gamma] \zeta_i \frac{\partial}{\partial \zeta_i} \Pi + \rho \left[d_i \zeta_i - (\sum_{j \in \mathcal{N}_i} \zeta_j) \right] \frac{\partial}{\partial \zeta_i} \Pi \right. \\
&+ \frac{1}{2} \left[\beta(1-\phi_i)\phi_i + \gamma\phi_i + \rho(d_i\phi_i + (1-\phi_i) \sum_{j \in \mathcal{N}_i} \phi_j) \right] \frac{\partial^2}{\partial \zeta_i^2} \Pi \\
&- \left. \rho\phi_i \sum_{j \in \mathcal{N}_i} (1-\phi_j) \frac{\partial^2}{\partial \zeta_i \partial \zeta_j} \Pi \right)
\end{aligned}$$

We assume that the mean-field solution is the same for all populations, $\phi_i = \phi$.

$$\begin{aligned}
\frac{\partial \Pi}{\partial t} &= \sum_{i=1}^{M^2} \Bigg(\left[\beta(1-2\rho)\phi + \gamma + d_i\rho\phi\right] \zeta_i \frac{\partial}{\partial \zeta_i}\Pi - \rho\Big(\sum_{j\in\mathcal{N}_i} \zeta_j\Big)\frac{\partial}{\partial \zeta_i}\Pi \\
&+ \frac{1}{2}\left[(\beta + 2d_i\rho)(1-\phi)\phi + \gamma\phi\right] \frac{\partial^2}{\partial \zeta_i^2}\Pi \\
&- \rho\phi(1-\phi) \sum_{j\in\mathcal{N}_i} \frac{\partial^2}{\partial \zeta_i \partial \zeta_j}\Pi \Bigg)
\end{aligned}$$

Then it follows in the form of the Fokker-Planck Equation 10:

$$\begin{aligned}
A_{ii} &= -(\beta(2\phi - 1) + \gamma + d_i\rho), \\
A_{ij} &= \rho, \text{ if } i \text{ and } j \text{ are adjacent}, \\
B_{ii} &= (\beta + 2\rho d_i)\phi(1-\phi) + \gamma\phi, \\
B_{ij} &= -2\rho\phi(1-\phi), \text{ if } i \text{ and } j \text{ are adjacent},
\end{aligned}$$

4 Metapopulation Model: simulations and predictions

4.1 Variance

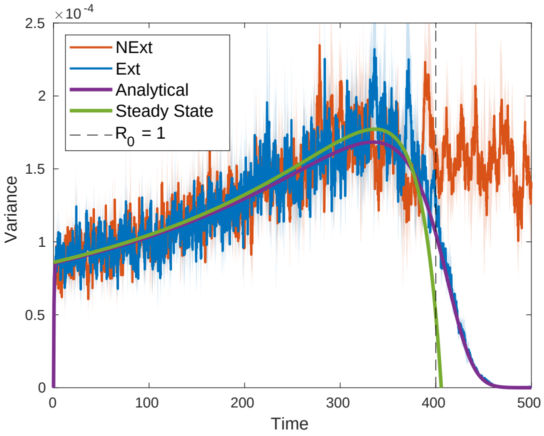

(a) 9 populations - Variance over 50 realisations

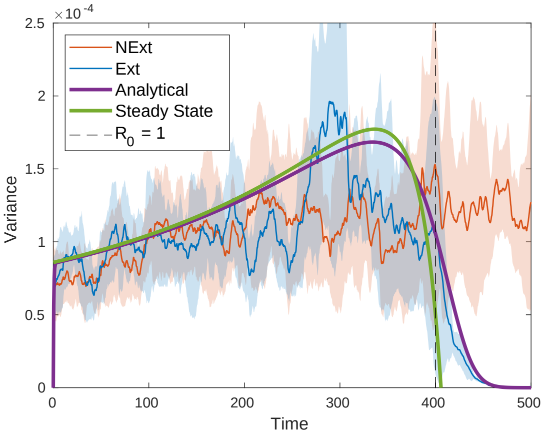

(b) 9 populations - Variance, one realisation

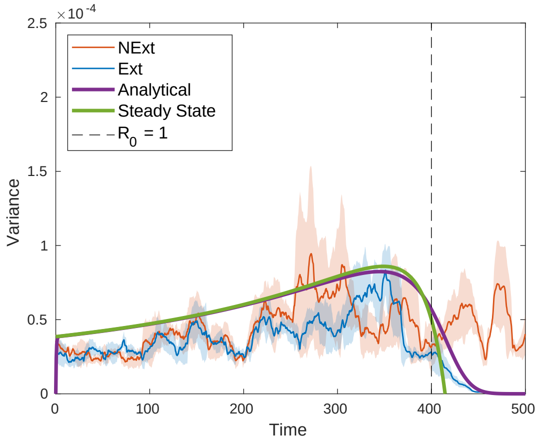

(c) 4 populations - Variance, one realisation

Figure 3: **Metapopulation:** Comparing predictions to simulations of the variance calculated: (a) over 50 realisations; (c), (b) by detrending using the mean of M^2 subpopulations before calculating variance over a moving window of size 200 timepoints (single realisation, averaged over all subpopulations). Each figure shows: steady state predictions (green line); dynamic predictions (purple line); simulations of the model going extinct (Ext, blue line) and simulations of the model not going extinct (NExt, red line). For repeated simulations each line is the mean value obtained over 50 simulations and the shaded area represents one standard deviation about the mean.

4.2 Parameter sensitivity analysis for movement rate and transmission in the metapopulation model

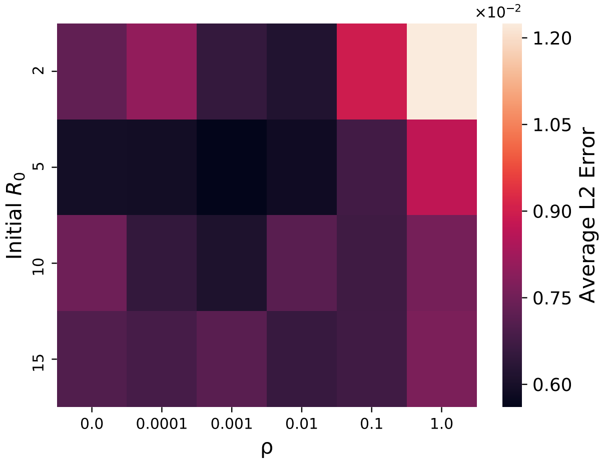

Figure 4: **Metapopulation**: heatmap assessing how the movement rate between populations (ρ) and the transmission rate (β) compare when calculating the L2 error between the analytical solution and simulations produced by the Gillespie algorithm. L2 Norm error between the analytical prediction and simulations of the variance calculated over realisations, after detrending using the mean of the subpopulations for $M = 3$.

4.3 Coefficient of Variation

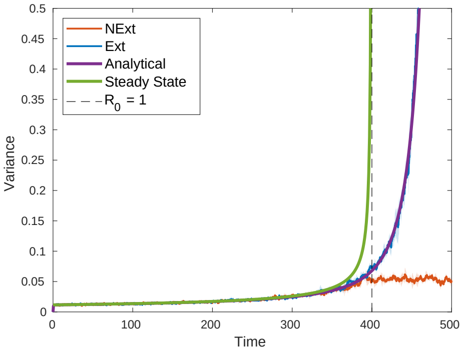

(a) 9 populations - CV over 50 realisations

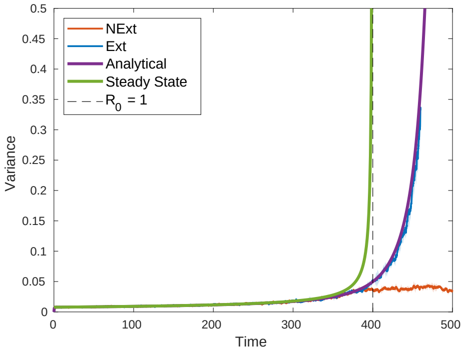

(b) 4 populations - CV over 50 realisations

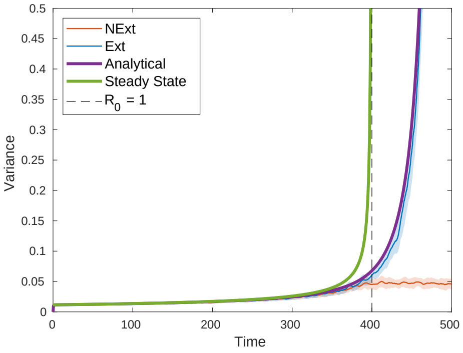

(c) 9 populations - CV, detrending between 9 subpopulations

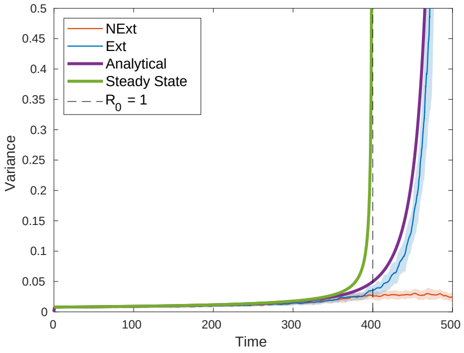

(d) 4 populations - CV, detrending between 4 subpopulations

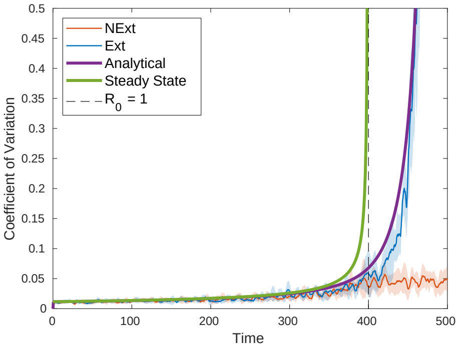

(e) 9 populations - CV, one realisation

Figure 5: **Metapopulation:** Comparing predictions to simulations of the CV calculated: (a), (b) over 50 realisations; (c), (b) by detrending using the mean of M^2 subpopulations before calculating CV over a moving window of size 50 timepoints (averaged over 50 realisations); and (e) by detrending using the mean of M^2 subpopulations before calculating CV over a moving window of size 200 timepoints (single realisation, averaged over all subpopulations)). Each figure shows: steady state predictions (green line); dynamic predictions (purple line); simulations of the model going extinct (Ext, blue line) and simulations of the model not going extinct (NExt, red line). For repeated simulations each line is the mean value obtained over 50 simulations and the shaded area represents one standard deviation about the mean.

4.4 ROC curve analysis

The ROC curves below were calculated using the Kendall's tau rank correlation coefficient. Kendall-tau coefficient is a test of statistical significance that is widely used in the literature for early warning signals of critical transitions. The statistic was calculated on a moving window for $50 * M^2$ realisations of NExt and Ext, for M^2 subpopulations. We calculated the Kendall-tau coefficient for each realisation as measure if the increasing trend in the variance and CV is statistically significant. We evaluated this for our simulations up to a variety of endpoints: $t = 400$ to $t = 450$. We considered different endpoints since the dynamics (and therefore the increasing trend) of NExt and Ext are the same up until $R_0 = 1$ at $t = 400$.

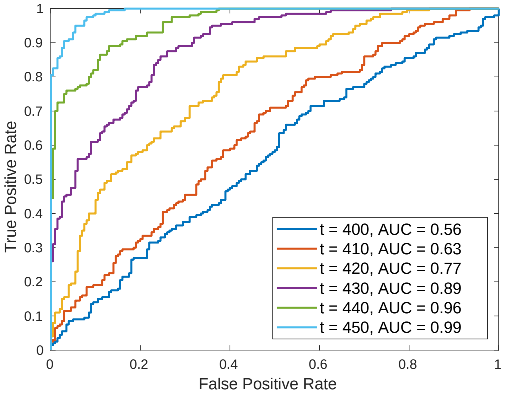

(a) 4 populations Variance - Kendall-tau ROC, detrending using mean of subpopulations

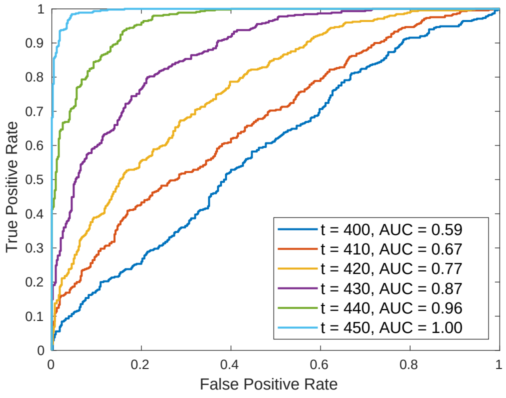

(b) 9 populations Variance - Kendall-tau ROC, detrending using mean of subpopulations

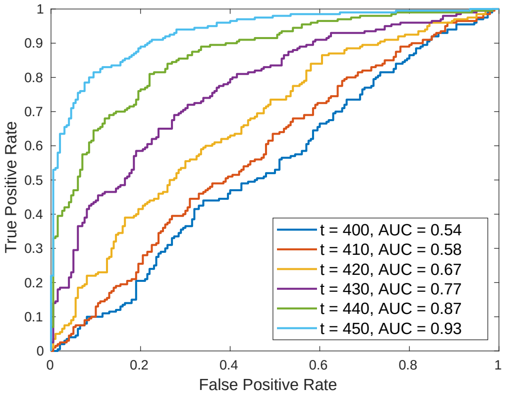

(c) 4 populations Variance - Kendall-tau ROC, detrending using 50 simulations

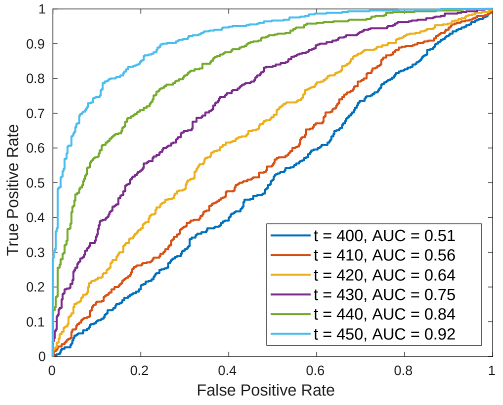

(d) 9 populations Variance- Kendall-tau ROC, detrending using 50 simulations

Figure 6: **Metapopulation:** ROC curves calculated over 50 realisations using Kendall's tau rank correlation coefficient for the timeseries up to various timepoints ((e),(f)). Each curve calculates the statistic on a window of size 50 timepoints after detrending: (c), (d) using the mean of M^2 subpopulations; and (e), (f) using the mean over 50 realisations.

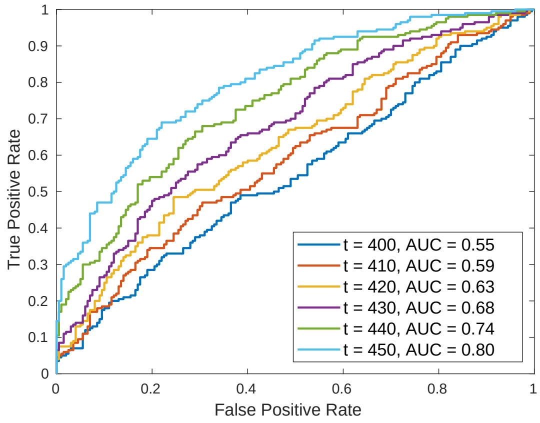

(a) 4 populations CV - Kendall-tau ROC, detrending using mean of subpopulations

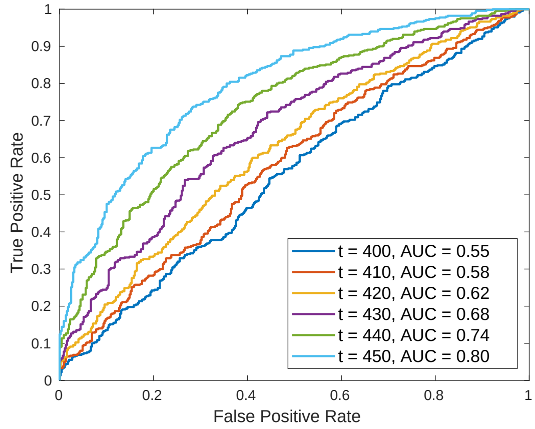

(b) 9 populations CV - Kendall-tau ROC, detrending using mean of subpopulations

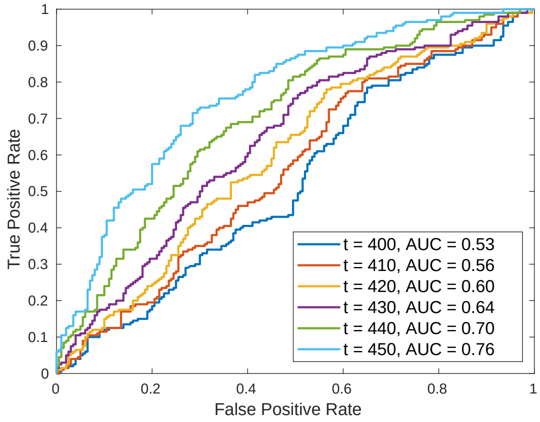

(c) 4 populations CV - Kendall-tau ROC, detrending using 50 simulations

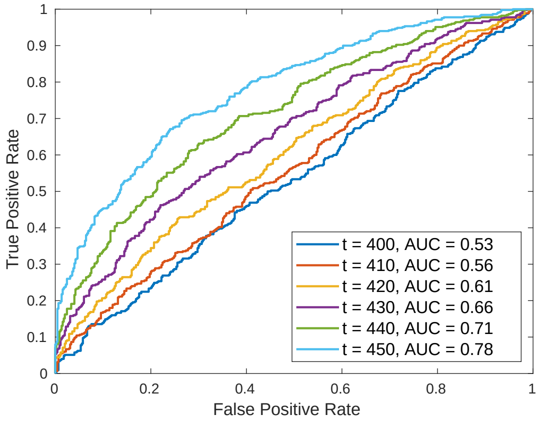

(d) 9 populations CV- Kendall-tau ROC, detrending using 50 simulations

Figure 7: **Metapopulation:** ROC curves calculated over 50 realisations using Kendall's tau rank correlation coefficient for the timeseries up to various timepoints. Each curve calculates the statistic on a moving window of size 50 timepoints after detrending: (a), (b) using the mean of M^2 subpopulations; and (c), (d) using the mean over 50 realisations.
